# Neural networks estimate muscle force in dynamic conditions better than Hill-type muscle models

**DOI:** 10.1242/jeb.250268

**Published:** 2025-11-18

**Authors:** Maria Eleni Athanasiadou, Monica A. Daley, Anne D. Koelewijn

**Affiliations:** ^1^ Chair of Autonomous Systems and Control, Department of Electrical Engineering, Friedrich-Alexander-Universität Erlangen-Nürnberg, 91052 Erlangen, Germany; ^2^ Department of Ecology and Evolutionary Biology, University of California, Irvine, Irvine, CA 92697, USA; ^3^ Biomedical Engineering & Mechanical and Aerospace Engineering, Henry Samueli School of Engineering, University of California, Irvine, Irvine, CA 92697-2700, USA

**Keywords:** Guinea fowl, Machine learning, Sonomicrometry, Electromyography, Force–length–velocity relationships, Dynamic muscle behavior

## Abstract

Hill-type muscle models are widely used, even though they do not accurately represent the relationship between activation and force in dynamic contractions. We explored the use of neural networks as an alternative approach to capture features of dynamic muscle function, without a priori assumptions about force–length–velocity relationships. We trained neural networks using an existing dataset of two guinea fowl muscles to estimate muscle force from activation, fascicle length and velocity. Training data were recorded using sonomicrometry, electromyography and a tendon buckle. First, we compared the neural networks with Hill-type muscle models, using the same data for network training and model optimization. Second, we trained neural networks on larger datasets, in a more realistic machine learning scenario. We found that neural networks generally yielded higher coefficients of determination and lower errors than Hill-type muscle models. Neural networks performed better when estimating forces on the muscle used for training, but on another bird, than on a different muscle of the same bird, likely due to inaccuracies in activation and force scaling. We extracted force–length and force–velocity relationships from the trained neural networks and found that both effects were underestimated and the relationships were not well replicated outside the training data distribution. We discuss suggested experimental designs and the challenge of collecting suitable training data. Given a suitable training dataset, neural networks could provide a useful alternative to Hill-type muscle models, particularly for modeling muscle dynamics in faster movements; however, scaling of the training data should be comparable between muscles and animals.

## Teaser

**Summary:** Neural networks predict muscle forces more accurately than Hill-type muscle models, particularly under dynamic conditions; however, they struggle to replicate the force–length and force–velocity relationships well.

## INTRODUCTION

The human musculoskeletal system has sparked scientific interest for centuries, as knowledge of internal body forces is crucial in clinical assessments (Steele et al., 2012), prosthesis design (Ferris et al., 2012) and injury prevention (Matijevich et al., 2019), among others. However, direct measurements of these forces require highly invasive sensors and procedures, and are therefore generally not performed in humans, especially not during movement. Instead, scientists have focused on the musculoskeletal system of other species to understand the mechanics of muscles and other tissues. A. V. Hill originally deciphered the inner workings of muscles using frog specimens at maximum activation (Hill, 1938). Based on his research, the so-called Hill-type muscle models have been developed, which describe muscle mechanics and can be used to simulate muscle behavior. Hill-type models are widely applied to answer research questions in humans (e.g. Steele et al., 2012; Koelewijn and Selinger, 2022) and other animals (e.g. Hill, 1938; Lee et al., 2013; Lemaire et al., 2016) by mathematically describing the observed relationship between force development, muscle length and muscle velocity. This relationship has since been expanded to include muscle activation (Hatze, 1977).

Hill-type muscle models originally consisted of two components: the contractile element (CE), representing the active muscle fiber and primarily capturing the force–length–velocity relationship (Zajac, 1989), and the series elastic element (SEE), representing the passive tissue in series – mainly the tendon and aponeurosis – and originally also including the compliance of fibers (Miller, 2018). The various shortcomings of the original models, such as the oversimplification of muscle behaviors or the lack of accuracy, have since been addressed by many different Hill-type model formulations that vary in complexity. Usually, complexity was increased in order to incorporate more complex muscle behaviors and thereby increase accuracy. The common addition of a parallel elastic element (PEE) (Miller, 2018), the integration of muscle fiber distribution (Herzog, 1999), muscle fiber recruitment (Lee et al., 2013) and orientation (Van den Bogert et al., 2011), dynamic length–tension relationships (Herzog, 1999) and muscle geometry and activation dynamics, which allowed force prediction under different physiological conditions (Zajac, 1989), are some of the most prominent adaptations to the original Hill-type models. Besides their accuracy, the computational efficiency of Hill-type models in large-scale simulations has also been addressed by employing implicit numerical methods (Van den Bogert et al., 2011).

Despite the widespread use and gradual evolution of Hill-type muscle models, it is known that these models still do not fully capture muscle behavior as recorded experimentally. The isometric force–length and isotonic force–velocity relationships are central components of Hill-type models. These relationships were formulated under tightly controlled experimental conditions, where length and tension were kept constant, respectively. Therefore, they rarely represent typical *in vivo* conditions, leaving room for uncertainty as to whether, or to what extent, they accurately reflect dynamic *in vivo* muscle behavior. Another important aspect of muscle mechanics that the Hill-type model does not capture is the tension change in muscle following a stretch or a shortening (Abbott and Aubert, 1952). It has been suggested that titin acts as a spring, creating force enhancement during active stretch and force depression during active shortening (Nishikawa et al., 2012). Other aspects that are not commonly included in the Hill-type muscle model are changes in optimal fiber length and maximum shortening velocity with activation level (Holt and Azizi, 2014, 2016). Roszek and Huijing (1997) and Rack and Westbury (1969) have shown that the optimal fiber length changes with the muscle activation level, while Chow and Darling (1999) showed the same for the maximum shortening velocity. These Hill-type muscle model limitations could be related to the fact that the model is applied to different activation levels, while it is based on experiments performed at maximum activation (Hill, 1938).

To overcome these limitations, in recent years, further extensions have been proposed to the Hill-type muscle model (e.g. Chow and Darling, 1999; McGowan et al., 2013; Millard et al., 2013), while completely new models have also been proposed (e.g. Tahir et al., 2018; Millard et al., 2024). For example, the history-dependent Hill-type model (McGowan et al., 2013) incorporates the force enhancement or depression caused by the muscle being stretched or shortened (Miller, 2018; Abbott and Aubert, 1952), respectively. Another extension includes the relationship between maximum shortening velocity and muscle stimulation (Chow and Darling, 1999; Nitschke et al., 2020). Instead of extending the Hill-type model, Whitney et al. (2022) and Millard et al. (2024) developed completely new phenomenological models to improve muscle model accuracy. These models are based on the mechanics of the three muscle filaments: actin, myosin and titin (Nishikawa et al., 2012; Millard et al., 2024), while Hill-type models only include the actin and myosin filaments. Both models (Millard et al., 2024; Whitney et al., 2022) outperform the Hill-type model, yet their force estimations could still be improved further (Millard et al., 2024), and setting their parameters can be difficult (Whitney et al., 2022).

Instead of further improving these existing phenomenological models, an alternative approach is to use machine learning to develop a model completely based on *in vivo* muscle mechanics data, gathered during different activities (Liu et al., 1999). The advantage of a neural network is that it does not require unverified assumptions about the model shape (Millard et al., 2024), as the network can replicate any function. In theory, a large and rich enough dataset containing dynamic data should enable us to train a neural network that replicates muscle mechanics relevant to movement and is more accurate than a model based on *ex vivo* experiments. In practice, obtaining a dynamic dataset on humans is virtually impossible, as sensors should be implanted into the muscles to record muscle length, force and activation. However, such datasets have been collected for guinea fowl (Daley and Biewener, 2011) and goats (Lee et al., 2013), among others. Similar to a Hill-type model, which is normalized to optimal fiber length and maximum isometric force to be applicable to different muscles and species, a neural network trained on such a dataset might also be applicable to other muscles and species, provided that such an input training dataset is available and the model is appropriately normalized. It should also be validated that the trained network correctly represents muscle mechanics. Previously, an artificial neural network was successfully trained to estimate muscle force from electromyography data of a cat soleus (Liu et al., 1999). However, the relationship between electromyography and muscle force is not always direct, as muscle length and velocity also affect force generation (Nishikawa et al., 2007). This relationship is especially visible in a dataset containing perturbations, such as in the dataset by Daley and Biewener (2011).

Therefore, in this paper, we developed neural networks to estimate muscle force from muscle length, velocity and activation. We trained those neural networks on existing data from guinea fowl (Daley and Biewener, 2011). First, we trained neural networks on a smaller dataset and compared their accuracy with that of a widely used Hill-type muscle model, which was optimized using the same data that was used to train the neural network. For this comparison, we chose a two-element Hill-type muscle model with only a force–length–velocity relationship, rather than a more complex model. Such complex models are commonly used to investigate muscle mechanics of individual muscles (Zahalak, 1986; McGowan et al., 2013). However, they are not necessarily more effective than Hill-type muscle models (Lemaire et al., 2016), which remain the norm because of their computational convenience and ease of parametrization (Wakeling et al., 2023). Thus, the comparison of neural networks with these widely used standards offers more practical insight than benchmarking against rarely used, more complex Hill-type muscle model variations. It also paves the way towards another modeling approach to better capture dynamic features of muscle contraction than current Hill-type muscle models. Second, we investigated the accuracy of networks that were trained on larger datasets and thus a more realistic machine learning scenario. We first evaluated the networks by comparing their estimated muscle forces with the measured ones and then explored whether the networks replicated known muscle mechanics, specifically the force–length and force–velocity relationships. We investigated whether these relationships emerge from the networks, which would mean that they correctly represent muscle mechanics as these are inferred from controlled experimental data. Therefore, this comparison could provide an additional validation of the neural networks' ability to fully capture muscle behavior, which is necessary to confidently use these models across species.

List of symbols and abbreviations

*a*
: muscle activation
*A*_rel_
: curvature of the force–velocity relationship
*c*_3_
: continuity factor ensuring a smooth transition in the force–velocity relationship
CE: contractile element
DF: digital flexor
*F*_CE_
: contractile element force
*F*_Hill_
: tendon or muscle force calculated by Hill-type model
*f*(*l*_CE_): force–length relationship
*F*_max_
: maximal isometric force
*F*­_meas_: experimentally measured tendon or muscle force
*F*_PEE_
: parallel elastic element force
*g*_max_
: maximum force amplification during muscle lengthening
*g*(*v*­_CE_): force–velocity relationship
Hill-r01: Hill-type muscle model optimized on the first trial of bird 1, at 1.8 m s^−1^ and a level surface
Hill-r12: Hill-type muscle model optimized on the twelfth trial of bird 1, at 4.5 m s^−1^ with 7 cm obstacles
*k*_PEE_
: stiffness of the parallel elastic element
*l*_CE_
: muscle or contractile element length
LG: lateral gastrocnemius
*l*­_slack,PEE_: slack length of the parallel elastic element
mRMSE: mean root-mean-square error
NN-b1: neural network trained on a large dataset with trials of varying obstacle heights (level, 5 and 7 cm) and speeds (1.8, 3.0, 3.5, 3.8 and 4.5 m s^−1^); training excluded all trials of bird 1
NN-b2: neural network trained on a large dataset with trials of varying obstacle heights (level, 5 and 7 cm) and speeds (1.8, 3.0, 3.5, 3.8 and 4.5 m s^−1^); training excluded all trials of bird 2
NN-b3: neural network trained on a large dataset with trials of varying obstacle heights (level, 5 and 7 cm) and speeds (1.8, 3.8 and 4.5 m s^−1^); training excluded all trials of bird 3
NN-b5: neural network trained on a large dataset with trials of varying obstacle heights (level, 5 and 7 cm) and speeds (1.8, 3.0, 3.5, 3.8 and 4.5 m s^−1^); training excluded all trials of bird 5
NN-r01: neural network trained on the first trial of bird 1, at 1.8 m s^−1^ and a level surface
NN-r12: neural network trained on the twelfth trial of bird 1, at 4.5 m s^−1^ with 7 cm obstacles
PEE: parallel elastic element
*R* ^2^
: coefficient of determination
RMSE: root-mean-square error
RMSE%: relative root-mean-square error
SEE: series elastic element
*v*_CE_
: muscle or contractile element velocity
*v*_CE,max_
: maximum shortening velocity of the force–velocity relationship
*W*
: width of the force–length relationship

## MATERIALS AND METHODS

We used an existing dataset that was recorded on guinea fowl, containing activation, muscle length and muscle velocity as inputs and muscle force as output. The dataset is available from Zenodo (https://doi.org/10.5281/zenodo.15968637). We used this dataset for network training and Hill-type muscle model optimization, and evaluated how well neural networks and Hill-type muscle models could estimate forces on the same muscle and bird as used for training, a different muscle of the same bird, the same muscle of a different bird, and a different muscle of a different bird. All code used for neural network training, Hill-type muscle model optimization and analysis is available from Zenodo (https://doi.org/10.5281/zenodo.15836088).

### Guinea fowl data

**Fig. 1.**
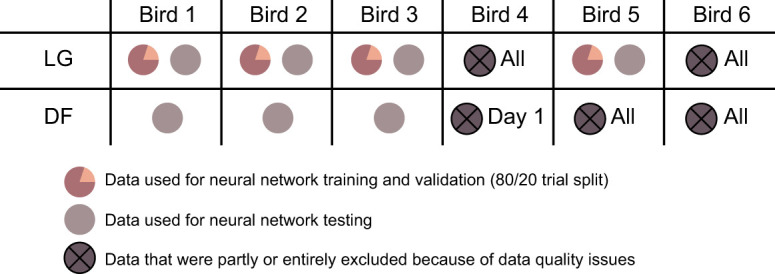
**Layout of the usability of the original data.** All trials of the lateral head of the gastrocnemius (LG) muscle for bird 4, the digital flexor (DF) muscle of bird 5, and both muscles of bird 6 were not usable. Trials gathered on day 1 for the DF muscle of bird 4 were also of poor quality.

The dataset contained data from six guinea fowl, *Numida meleagris* (Linnaeus 1758). We summarize the protocol here and refer readers to Daley and Biewener (2011) for further details. Briefly, this dataset contained between 12 and 15 walking and running trials of about 15 to 30 s for each bird. The measurements were taken during level and obstacle trials between 1.8 and 4.5 m s^−1^. During the obstacle trials, the birds would suddenly step on a raised obstacle (5 or 7 cm) every four to five steps. Data were recorded using a tendon buckle (force), sonomicrometry (contractile element length) and electromyography (activation) for two different muscles; the lateral head of the gastrocnemius (LG) and the digital flexor to the lateral toe (DF). The contractile element velocity in the dataset was calculated as the time derivative of the CE length. Out of the six guinea fowl, we used the data of both muscles for three birds. For the fourth bird, the data quality of the LG was not very good as a result of cross-talk noise in the electromyography, and the quality of the sonomicrometry for the DF was poor on day 1. Therefore, for bird 4 we did not include the DF data recorded on day 1 and the LG data in its entirety. For the fifth bird, we only used the LG data, as the electromyography failed for the DF. We omitted the sixth bird entirely because of data quality issues (Fig. 1). In previous studies (e.g. Daley and Biewener, 2011), a sufficient sample of individual strides with good data quality were identified for each bird, but this approach is not suitable here, because training neural networks requires as much data as possible and thus entire trials and consecutive strides rather than individually picked gait cycles.

We used the tendon force in N, the normalized CE length and velocity, and the raw electromyography signal and processed the data as follows. First, we computed the maximum isometric force of each bird's LG and DF using the physiological cross-sectional area (Daley and Biewener, 2011), and a maximum stress of 0.29 N mm^−2^, as reported by Schwaner et al. (2024) for guinea fowl (see table 1 of Schwaner et al., 2024). This maximum stress value also falls within the commonly accepted range of 0.26–0.4 (Ker et al., 1988; Wells, 1965; Jayes and Alexander, 1982; Head et al., 2011). We then normalized the measured tendon force using this maximum isometric force. Then, we processed the electromyography data similar to Whitney et al. (2022): we first applied a third-order Butterworth high-pass digital filter in zero-phase, via forward and backward filtering, each with a cut-off frequency of 30 Hz. Then we rectified the signal, after which we applied a third-order low-pass digital filter, again in zero phase, with a cut-off frequency of 6 Hz. This activation signal was then normalized to the average peak measured activation found in the filtered electromyography data. Eight average peak measured activation values were calculated, one for each muscle of each bird. The peaks were detected using the filtered activation signal of the highest-speed trials of each bird and muscle. The threshold for peak detection was 0.1 for all birds and muscles, except for the DF of bird 3, where we used 0.035. A time delay of 23.6 ms was added to the activation to account for excitation–contraction coupling (Daley and Biewener, 2011). The same 6 Hz low-pass filter was also applied to the CE velocity.

### Neural network training

We designed neural networks to estimate tendon force from activation, CE length and CE velocity. To replicate a Hill-type muscle model as close as possible, without introducing any bias in favor of the neural networks (e.g. by incorporating history dependence), these networks were set up to calculate the tendon force at each time point individually from the inputs at the same time point. First, we compared two neural network types with the Hill-type muscle model and trained them on the same data that was also used to optimize the Hill-type muscle model parameters. Because of the computational cost related to the model optimization, we made this comparison on a smaller dataset, consisting of a single trial. Specifically, we trained the first network type (NN-r01) using the data of the LG of the first trial of bird 1, a level trial at 1.8 m s^−1^. The second network type (NN-r12) was trained on the twelfth trial of the same bird and muscle, a trial with 7 cm obstacles at 4.5 m s^−1^. Second, we trained neural networks on larger datasets to investigate their prediction accuracy in a more commonly used machine learning scenario. We used a leave-one-out training approach and trained four networks, excluding the LG data of a different bird for each, so that each network was trained on the LG data of three birds. We call these network types NN-b1 (bird 1 was left out), NN-b2 (bird 2 was left out), NN-b3 (bird 3 was left out) and NN-b5 (bird 5 was left out). The number of trials used for training ranged from 39 to 41.

We used the following process to train the networks. First, we split the data into three categories; the training data, on which the neural network was trained, the validation data, to validate the hyperparameter optimization, and the test data, on which the trained neural network made predictions. The training data contained the first 80% of all trials used for training, while the validation was performed on the last 20%. This split was chosen to ensure that the training data did not include information from a later time point than any of the validation data, which could lead to data leakage. We normalized the CE length and velocity to have a mean of zero and a standard deviation of one. We optimized the hyperparameters, specifically the number of hidden layers, number of nodes in each layer and the activation function of the hidden layers, using the automatic hyperparameter optimization in MATLAB's fitrnet, which uses a Bayesian optimization. We repeated this hyperparameter optimization 10 times when training on a single trial and 5 times when training on the large dataset to account for random effects. We performed all training and analysis in MATLAB R2022b and R2024b (MathWorks, Natick, MA, USA).

### Hill-type muscle model and optimization

We used the Hill-type muscle model described by Zajac (1989), Van den Bogert et al. (2011) and Herzog (1999). We chose this model as its mathematical equations are such that the shape of the force–length and force–velocity relationship is preserved during optimization. We found that for other models, optimizing the equations could change the shape of the force–length and force–velocity relationships. Furthermore, preliminary comparisons showed that this Hill-type model predicted muscle forces with higher correlation and lower errors than the models by Wakeling et al. (2012), Russell Esposito and Miller (2018), De Groote et al. (2016), Geyer and Herr (2010), Thelen (2003) and Millard et al. (2013).

We determined the tendon force, *F*_Hill_, as the sum of the force in the contractile element, *F*_CE_, and the parallel elastic element, *F*_PEE_, using the following relationships:
(1)

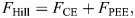
with
(2)

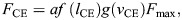

(3)

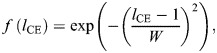

(4)

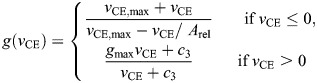

(5)

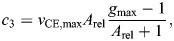

(6)

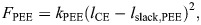
where *a* is muscle activation; *F*_max_ is the maximal isometric force; *f*(*l*_CE_) is the force–length relationship with width, *W*; *g*(*v*_CE_) is the force–velocity relationship with maximum shortening velocity, *v*_CE,max_, curvature of the force–velocity relationship, *A*_rel_, maximum force amplification during lengthening, *g*_max_, and factor *c*_3_; *k*_PEE_ is the stiffness of the parallel elastic element; and *l*_slack,PEE_ is the slack length of the parallel elastic element. The factor *c*_3_ was calculated such that the force–velocity relationship is continuous. Note that the muscle length, *l*_CE_, and velocity, *v*_CE_, are normalized to optimal fiber length, *L*_0_.

We optimized the Hill-type muscle model using the same data as used for network training, to allow for a fair comparison between the model and the neural network. We optimized the width of the force–length relationship, *W*, the maximum shortening velocity, *v*_CE,max_, the curvature of the force–velocity relationship, *A*_rel_, the maximum force amplification during lengthening, *g*_max_, the stiffness of the parallel elastic element, *k*_PEE_, and its slack length, *l*_slack,PEE_. As an objective function, we used the mean square error between the calculated (*F*_Hill_) and measured muscle force. We optimized the parameters using covariance matrix adaptation, evolutionary strategy (CMA-ES) (Hansen and Ostermeier, 2001), using 100 iterations. We performed the optimizations in MATLAB R2022b (MathWorks).

### Data analysis

**Fig. 2.**
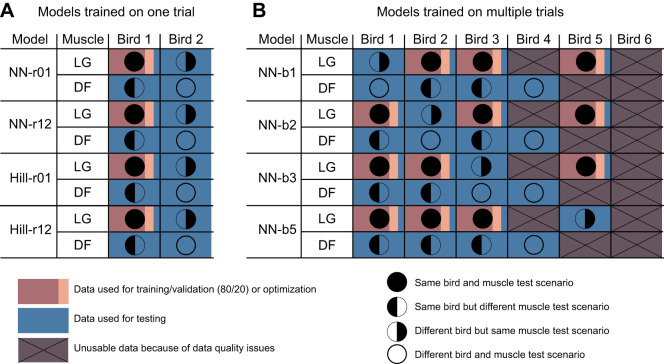
**Layout of data use for all Hill-type muscle models and network types.** (A) The approach comparing neural networks with Hill-type muscle models trained on the lateral head of the gastrocnemius (LG) muscle of bird 1 with trial 1 or 12 (NN-r01, NN-r12, Hill-r01 and Hill-r12). (B) The approach for the neural networks trained on a larger dataset (NN-b1 to NN-b5, where ‘bX’ indicates the bird reserved for testing). The colors show data use, where the training and validation data were split in each trial, and the circles the test scenario. DF, digital flexor muscle.

We performed two analyses to investigate neural networks as muscle models. First, we investigated whether a neural network can outperform a Hill-type muscle model. To do so, we optimized the Hill-type muscle model and trained the neural networks on the same data of bird 1. We trained two neural networks and optimized the Hill-type muscle model twice: once using the first trial at 1.8 m s^−1^ and a level surface and once using the twelfth trial at 4.5 m s^−1^ with 7 cm obstacles (Fig. 2). We call these models NN-r01, NN-r12, Hill-r01 and Hill-r12, respectively. As the width of the force–length relationship is muscle specific, we performed two additional optimizations, one starting from Hill-r01 and one starting from Hill-r12, using data from the DF muscle, in which only the width was optimized based on the DF muscle data of the same trials. Second, we investigated the accuracy of neural networks in a more common training approach using larger datasets. We trained on the LG data of all but one bird, for which good quality data were available, and on all but one trial of the included birds, and tested on the remaining trial for each bird and all trials of the excluded bird, as well as on all good quality data of the DF (Fig. 2). We call these models NN-b1, NN-b2, NN-b3 and NN-b5, indicating which bird was excluded for testing.

We compared all models using six metrics, and added a seventh for models NN-b1, NN-b2, NN-b3 and NN-b5. We used the (1) mean root-mean-square error (mRMSE) between measured and estimated forces, normalized to maximum isometric force, (2) the median coefficient of determination between measured and estimated forces, the mean percentage of (3) missed force peaks and of (4) extra force peaks (peaks which were spuriously generated), (5) the mRMSE of the rise time between measured and estimated forces and (6) the mRMSE of the fall time between measured and estimated forces. The mean and standard deviation or median and range were determined over all trials in the test data and, in the case of neural networks, over the training repetitions to account for variability in the Bayesian optimization. To find the percentage of missed and extra force peaks, we first detected peaks using a threshold equal to the mean value of the force after removing any baseline offset, plus 1.5 times the standard deviation. We then identified whether estimated and measured peaks aligned. To do so, we defined peaks to be aligned if the difference in location between the estimate and measurement was less than 45% of the 25th percentile of peak location differences in the trial. When multiple peaks were detected within this threshold, the closest to the measured one was used. The rise and fall time were defined as the time interval from 50% peak force to peak force and from peak force to 50% peak force, respectively. A seventh metric, the mean relative RMSE, was evaluated for the neural networks that were trained on larger datasets to facilitate comparison to previous research. The mean relative RMSE was calculated using the maximum measured force during each trial. We compared all models' prediction accuracy on four scenarios: on the same bird and muscle as used in training, on the same bird but another muscle (DF), on the same muscle of a different bird, and on another muscle (DF) of a different bird (Fig. 2).

Third, we compared the force–length and force–velocity relationships that emerged from the neural networks trained on larger datasets with those of a typical Hill-type muscle model, to investigate whether these networks could replicate muscle mechanics. To do so, we varied the fiber length between 60% and 140% of the optimal fiber length and calculated the resulting force normalized to maximum isometric force, assuming isometric conditions. The force–length relationship includes the PEE, as the CE and the PEE cannot be separated in a neural network. Then, we varied the CE velocity between −10 and 10 fiber lengths per second, assuming optimal fiber length, and again calculated the resulting force normalized to maximum isometric force. We repeated these calculations for five different activation levels: 0.2, 0.4, 0.6, 0.8 and 1.

## RESULTS

### Comparison of neural network with Hill-type muscle model

#### Neural network and Hill-type muscle model parameters

**Fig. 3.**
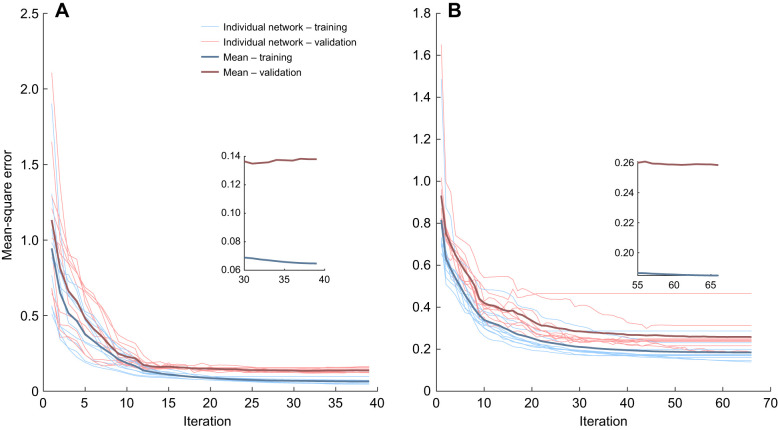
**Training and validation losses during training of neural networks NN-r01 and NN-r12.** The thin blue and red lines show the training and validation loss trajectories of individual neural networks of each type for (A) NN-r01 (level at 1.8 m s^−1^) and (B) NN-r12 (7 cm incline at 4.5 m s^−1^). The darker single blue and red lines show the average trend for each respective loss. The insets show an expanded view of the last 9 (A) or 11 (B) iterations.

We found that the training and validation losses for NN-r01 were lower than those for NN-r12, while for both models, the training and validation losses showed a similar trend (Fig. 3). After training, the best variant of neural network NN-r01 had a training loss of 0.052 and a validation loss of 0.13 after 32 iterations, while for NN-r12 the training loss was 0.16 and the validation loss 0.22 after 49 iterations. The models consisted of hidden layers with rectified linear activation units. The best variant of NN-r01 had two hidden layers, each with 71 and 41 nodes, while NN-r12 had three hidden layers, each with 35, 16 and 14 nodes, respectively. We also found that even very different network architectures, found during hyperparameter optimization, could result in similar losses and thus comparable performance. For example, for NN-r12, the number of hidden layers varied between one and three. The first of those layers ranged from 12 to 154 nodes, the second ranged between 5 and 244 nodes and the third between 9 and 285 nodes. Additionally, Fig. 3A may reflect early signs of overfitting, with the training loss gradually decreasing and the validation loss tending to plateau.

The optimized variables for Hill-r12 matched commonly reported values closer than the optimized variables for Hill-r01, while they both fitted the data equally well in the optimization. The final objective values of the Hill-type model optimization were 0.0004 and 0.0005 for Hill-r01 and Hill-r12, respectively. These values cannot be compared with the neural network losses, as the loss function was different from the optimization objective. The curvature of the force–velocity relationship (*A*_rel_) for Hill-r12 (0.23) was closer to the value range suggested by Hill (0.25–0.3) (Hill, 1938) than that of Hill-r01 (0.0278), and so was the width of the force–length relationship (*W*), which was 0.09 for Hill-r12 and 0.06 for Hill-r01, compared with the range of 0.1–0.3 (Schwaner et al., 2024). Both models' maximum shortening velocity values (*v*_CE,max_) were outside the range reported by Schwaner et al. (2024) for guinea fowl (5.6–8.6), with 4.56 and 10.21 *L*_0_ s^−1^ for Hill-r12 and Hill-r01, respectively. However, higher values have also been observed for larger birds (Nelson et al., 2004). Maximum force amplification during lengthening (*g*_max_) was 1.04 for Hill-r12 and 0.97 for Hill-r01, which did not match the values typically reported in the literature (1.5–1.8) (Tomalka, 2023) possibly because of the lack of active lengthening data in the measurements.

We found that re-optimizing the width of the force–length relationship based on the DF data did not alter our findings. The optimization led to a median coefficient of determination of −0.30 on bird 1 and −0.85 on bird 2 for trial 1, and −0.58 on bird 1 and −0.78 on bird 2 for trial 12, which was an improvement of less than 20% compared with Hill-r01 and Hill-r12. These coefficients of determination were still lower than those of the neural networks. All other metrics were higher than or comparable to Hill-r01 and Hill-r12. For example, the percentage of extra peaks for the DF increased from 2.8% to 3.1% for bird 1 and from 3.5% to 4.2% for bird 2 compared with Hill-r01, while the rise time mRMSE for the DF changed from 0.01 s for bird 1 and 0.03 s for bird 2 using Hill-r12 to 0.02 s for both birds. For these examples, both the original Hill-r01 and Hill-r12 and these re-optimized models performed better than the neural networks.

#### Neural network and Hill-type muscle model performance

**Fig. 4.**
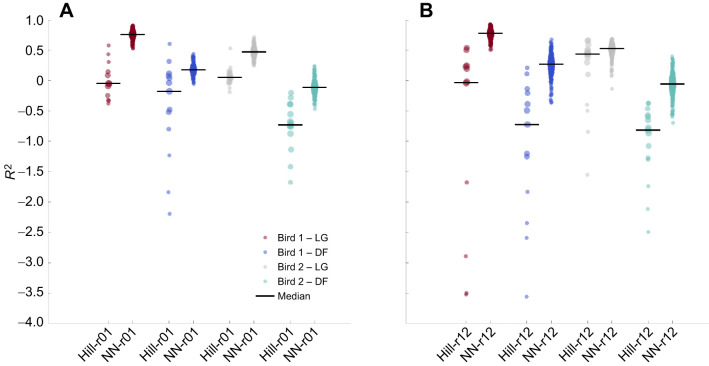
**Distribution of coefficients of determination for comparison between Hill-type muscle models and neural networks.** (A) Models fitted on trial 1. (B) Models fitted on trial 12. The black horizontal line shows the median for each model and test scenario. Data density is represented by the dot diameter and opacity. Hill-type muscle model distributions include all test trials, while neural network distributions include all test trials and all 10 network type variants.

Our neural networks outperformed Hill-type models on muscle force prediction, yielding lower mRMSEs and higher median coefficients of determination, and also outperformed on peak detection (Table 1). A lower mRMSE and a higher coefficient of determination were observed in all four test scenarios: when testing on the bird and muscle that were used for training and optimization, when testing on a different muscle, on a different bird, and when both were different (Table 1). The error reduction achieved by both the neural networks was at least 25% for the LG and 20% for the DF. Neural network NN-r12 had the lowest mRMSE between the two neural networks on all four test scenarios. For the Hill-type muscle models, the mRMSE of Hill-r01 was lower than that of Hill-r12, when tested on the same bird as used for optimization. In the two remaining scenarios, Hill-r12 performed better than Hill-r01 (Table 1). The median coefficients of determination of NN-r12 were the highest regardless of test scenario. For NN-r01, the lower spread of the coefficients of determination (Fig. 4) for the LG muscle predictions further suggests possible overfitting to the training muscle. For the Hill-type models, their mean coefficients of determination differed depending on the muscle. Hill-r01 outperformed Hill-r12 on the DF muscle and Hill-r12 outperformed Hill-r01 on the LG. Also, in all test scenarios, except when predicting on a different bird and the same muscle, their median coefficients of determination were negative. Conversely for neural networks, their coefficients of determination became negative only when predicting on a different muscle and bird. Also, in all test scenarios, the neural networks matched the existing measured force peaks much better than the Hill-type muscle models. The neural networks predicted fewer extra force peaks on the LG muscle compared with the Hill-type muscle models, whereas Hill-r12 predicted the least amount of extra peaks on the DF muscle.

**Table 1.** Metrics, aggregated over all trials and networks, between measured and estimated forces for each model and test scenario used to compare neural networks with Hill-type muscle models

Model	Bird	Muscle	Force mRMSE	Median *R*^2^ [min. max.]	Missed peaks [min. max.] (%)	Extra peaks [min. max.] (%)	Rise time mRMSE (s)	Fall time mRMSE (s)
Hill-r01	Bird 1	LG	0.03±0.005	−0.05 [−0.38 0.58]	4.4 [0 24.4]	6.7 [0 16.2]	0.02	0.02
Hill-r12			0.04±0.02	−0.03 [−4.12 0.54]	11.9 [1.5 33.3]	6.1 [0 14.1]	**0.005**	0.009
NN-r01			0.01±0.003	0.76 [0.53 0.91]	1.0 [0 7.3]	6.3 [0 12.2]	0.01	0.007
NN-r12			**0.01**±0.003	**0.78** [0.51 0.93]	**0.9** [0 7.0]	**5.9** [0 12.0]	0.009	**0.006**
Hill-r01	Bird 1	DF	0.12±0.02	−0.18 [−2.20 0.60]	18.3 [2.5 35.6]	2.8 [0 11.5]	0.02	0.04
Hill-r12			0.15±0.02	−0.72 [−3.55 0.21]	11.1 [2.5 18.0]	**2.7** [0 8.3]	**0.01**	**0.01**
NN-r01			0.10±0.02	0.18 [−0.05 0.44]	**1.3** [0 8.0]	7.8 [0 19.0]	0.04	0.03
NN-r12			**0.09**±0.02	**0.27** [−0.36 0.67]	2.7 [0 14.8]	5.7 [0 23.3]	0.03	0.02
Hill-r01	Bird 2	LG	0.06±0.01	0.05 [−0.18 0.53]	7.1 [0 26.5]	2.8 [0 8.8]	0.008	0.03
Hill-r12			0.05±0.01	0.44 [−1.55 0.66]	6.5 [0 18.9]	1.3 [0 6.3]	**0.006**	0.02
NN-r01			0.04±0.008	0.47 [0.25 0.72]	**0.8** [0 8.1]	**1.1** [0 8.1]	0.02	0.009
NN-r12			**0.04**±0.006	**0.53** [−0.13 0.69]	**0.8** [0 8.1]	1.3 [0 8.1]	0.01	**0.009**
Hill-r01	Bird 2	DF	0.15±0.05	−0.73 [−5.62−0.20]	22.6 [2.9 52.6]	3.5 [0 17.1]	0.02	0.04
Hill-r12			0.15±0.02	−0.82 [−2.49−0.37]	13.5 [0 37.1]	**3.1** [0 16.7]	**0.01**	0.03
NN-r01			0.11±0.02	−0.11 [−0.47 0.24]	**1.2** [0 8.6]	5.2 [0 17.8]	0.03	0.04
NN-r12			**0.11**±0.02	**−0.05** [−0.69 0.40]	3 [0 14.3]	4.8 [0 17.8]	0.03	**0.02**

Bold indicates the highest metric in each test scenario. NN, neural network; mRMSE, mean root-mean-square error; LG, lateral head of gastrocnemius; DF, digital flexor. Force mRMSE is given ±s.d.

Despite the neural networks outperforming the Hill-type models on the force mRMSE, the median coefficient of determination, the average percentage of missed force peaks and the mRMSE for the force fall time, they led to poorer estimates in the force rise time. For all test scenarios, Hill-r12 generated lower mRMSE estimates, compared with all other models. Neural networks performed better than or equivalent to Hill-type models when estimating the force fall time for all test scenarios except when predicting for the same bird but a different muscle. In this scenario, Hill-r12 outperformed all other models. Hill-type muscle models also predicted fewer extra peaks when making predictions on a different muscle.

**Fig. 5.**
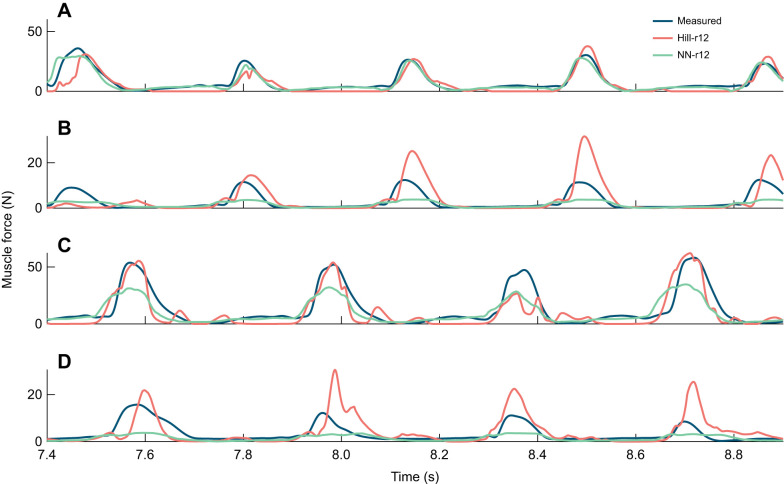
**Muscle force over time for part of a trial with speed 3.8 m s^−1^ and 7 cm elevation for the four test scenarios for the best performing neural networks and Hill-type muscle models (i.e. NN-r12 and Hill-r12).** (A) Same bird (bird 1), same muscle (LG). (B) Same bird (bird 1), different muscle (DF). (C) Different bird (bird 2), same muscle (LG). (D) Different bird (bird 2), different muscle (DF).

We found that the neural network estimations matched the measurements for all test scenarios, but especially well on the same bird and same muscle, indicating that there could be some overfitting to the training data (Fig. 5). Especially in Fig. 5A, the neural network's force estimates matched the measured data very well, while the low spread and better estimates of *R*^2^ for NN-r12 in this test scenario (Fig. 4) could indicate a propensity to overfit. The peak force in the DF muscle was underestimated by the neural network, while the Hill-type muscle model commonly overestimated this muscle's force by approximately 3 times the mean value of the measured force (Fig. 5B,D). Such a significant overestimation or even underestimation of force, in which prediction errors exceed the variability of measured values, would explain negative values in the coefficient of determination. At the same time, the duration of the individual force peaks tends to be longer in the neural network estimates than in the measurements. For the LG muscle of a different bird, the Hill-type muscle model estimates were less smooth than the measurements and the neural network estimates, but the magnitudes of the peaks matched better, and so did the timing of force generation. In addition, the Hill-type model produced overall narrower force peaks, compared with the neural network, leading to more accurate force rise time estimates (Fig. 5C).

### Predictions with neural networks trained on large datasets

The neural networks trained on large datasets combining different birds had training losses that ranged from 0.21 to 0.32 and validation losses that ranged from 0.20 to 0.26 after 42–140 iterations. The validation losses of these networks were close to those of NN-r01 and NN-r12, while the training losses were higher than that of NN-r01 and closer to that of NN-r12. All of the networks trained on large datasets consisted of one to three hidden layers with rectified linear activation units, except for NN-b3, which consisted of two hidden layers. The networks' architectures varied, with the first hidden layer ranging from 11 to 300 nodes, the second from 9 to 296 nodes and the third from 9 to 296 nodes. The difference between the training and the validation losses was smaller using the large dataset than using the smaller datasets, and they plateaued with a similar trend, which indicates a smaller likelihood of overfitting (Fig. S1).

**Fig. 6.**
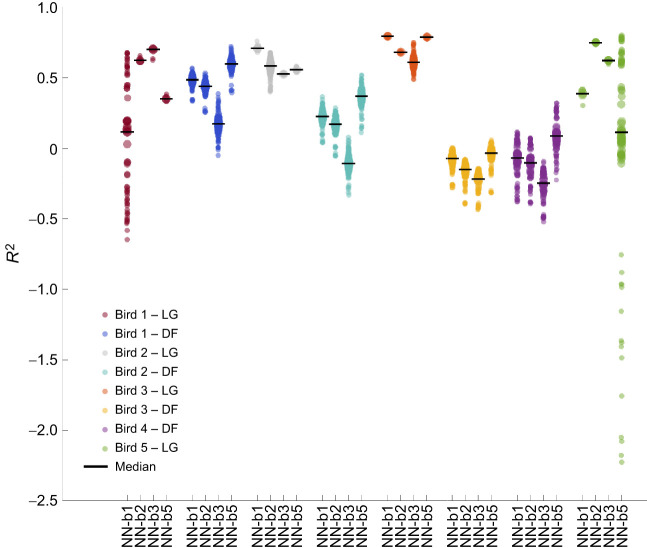
**Distribution of coefficients of determination for neural networks trained on a large dataset.** The black horizontal line shows the median. Data density is represented by the dot diameter and opacity. The neural network distributions include all test trials for the given bird and muscle, and all five network type variants. Note that this yields only five data points for the muscles and birds included in training, but 120 data points for the other scenarios.

Our networks trained on large datasets predicted muscle force more accurately when predicting on the same muscle of a different bird compared with predicting on a different muscle of the same or a different bird. Predictions on the LG resulted in lower mean relative RMSE, higher median coefficient of determination, and improved similarity in peaks and force rise and force fall times than predictions on the DF (Table 2). NN-b3 and NN-b5 were the least accurate when predicting on a different bird and muscle, as the majority of metrics on the DF of unknown birds (bird 4 for NN-b5, and birds 3 and 4 for NN-b3) were worse than those of the other birds. For NN-b1 and NN-b2, no significant difference in prediction accuracy was observed between birds excluded and included in training. For all networks, predictions on the DF of bird 3 and 4 led to worse median coefficients of determination, while their range was similar to the other bird and muscle combinations (Fig. 6). Most of these median coefficients of determination were negative, with the only exception being NN-b5 and the DF of bird 4, which resulted in a median coefficient of determination close to zero. Predictions on the DF muscle of the same two birds also generated more extra force peaks, compared with predictions on the DF of other birds (Table 2). However, NN-b1 and NN-b5 resulted in lower median *R*^2^ and a larger spread, when predicting on the LG of birds 1 and 5, respectively (Fig. 6).

**Table 2.** Metrics, aggregated over all trials and networks, between measured and estimated forces for each bird–muscle subgroup using the neural networks trained on large datasets

Model	Bird	Muscle	Force mRMSE	Force mRMSE (%)	Median *R*^2^ [min. max.]	Missed peaks [min. max.] (%)	Extra peaks [min. max.] (%)	Rise time mRMSE (s)	Fall time mRMSE (s)
NN-b1	Bird 1	LG	0.03±0.01	17.9	0.12 [−0.65 0.68]	3.38 [0 13.04]	4.86 [0 13.04]	0.013	0.016
	Bird 2		0.03±0.002	15.04	0.71 [0.68 0.76]	**0** [0 0]	**0** [0 0]	0.009	**0.004**
	Bird 3		0.02±0.0003	**7.91**	**0.79** [0.78 0.79]	**0** [0 0]	2.04 [0 9.43]	0.004	0.034
	Bird 5		**0.02**±0.0006	18.05	0.39 [0.30 0.41]	**0** [0 0]	**0** [0 0]	**0.003**	0.005
	Bird 1	DF	**0.08**±0.01	**17.30**	**0.48** [0.33 0.57]	**0.47** [0 5]	7.46 [0 18.18]	0.034	**0.022**
	Bird 2		0.10±0.02	17.72	0.22 [0.04 0.36]	1.04 [0 6.25]	**4.94** [0 17.78]	0.036	0.025
	Bird 3		0.26±0.03	25.07	−0.07 [−0.28 0.01]	2.13 [0 10.71]	11.49 [2.17 22.64]	0.013	0.037
	Bird 4		0.17±0.03	22.42	−0.07 [−0.38 0.11]	8.77 [0 25]	30.35 [7.69 62.50]	**0.012**	0.029
NN-b2	Bird 1	LG	0.02±0.0004	**8.12**	0.62 [0.61 0.66]	2.90 [1.45 4.35]	10.67 [10.53 10.81]	0.019	0.10
	Bird 2		0.04±0.01	14.97	0.58 [0.40 0.68]	0.5 [0 8.11]	1.16 [0 8.11]	0.017	**0.007**
	Bird 3		0.02±0.0002	9.78	0.68 [0.67 0.69]	**0** [0 0]	0.21 [0 1.03]	0.005	0.048
	Bird 5		**0.01**±0.0002	11.55	**0.75** [0.73 0.75]	0.78 [0 1.96]	**0** [0 0]	**0.002**	0.013
	Bird 1	DF	**0.08**±0.02	**18.07**	**0.44** [0.26 0.53]	**0.44** [0 5]	8.55 [0 18.6]	0.025	0.023
	Bird 2		0.10±0.02	18.37	0.17 [−0.06 0.28]	0.81 [0 5.71]	**5.16** [0 17.78]	0.029	**0.021**
	Bird 3		0.27±0.03	25.98	−0.15 [−0.40−0.08]	1.09 [0 7.14]	12.56 [2.17 27.1]	0.029	0.022
	Bird 4		0.17±0.03	22.64	−0.10 [−0.40 0.07]	3.56 [0 18.18]	29.88 [7.69 62.50]	**0.012**	0.029
NN-b3	Bird 1	LG	0.02±0.001	**7.61**	**0.70** [0.62 0.71]	8.41 [5.80 10.14]	9.71 [7.46 10.96]	0.019	0.004
	Bird 2		0.04±0.0002	19.44	0.53 [0.52 0.53]	**0** [0 0]	**0** [0 0]	**0.005**	**0.003**
	Bird 3		0.03±0.004	14.37	0.61 [0.49 0.75]	0.5 [0 2.56]	0.61 [0 3.85]	0.014	0.034
	Bird 5		**0.01**±0.0002	14.22	0.62 [0.60 0.62]	1.18 [0 1.96]	**0** [0 0]	0.007	0.028
	Bird 1	DF	**0.10**±0.01	21.71	**0.17** [−0.05 0.39]	**0.38** [0 2.78]	8.47 [0 18.18]	0.019	0.025
	Bird 2		0.11±0.02	**21.06**	−0.11 [−0.33 0.08]	1.29 [0 6.38]	**4.91** [0 17.78]	0.013	0.028
	Bird 3		0.28±0.03	26.68	−0.22 [−0.43−0.15]	1.54 [0 10.71]	11.55 [2.17 27.27]	**0.009**	**0.021**
	Bird 4		0.19±0.03	24.19	−0.25 [−0.52−0.09]	2.94 [0 13.64]	29.15 [7.69 60.61]	0.014	0.031
NN-b5	Bird 1	LG	0.02±0.0004	10.70	0.35 [0.33 0.38]	16.81 [13.04 26.09]	6.82 [3.39 7.81]	0.017	0.013
	Bird 2		0.04±0.0009	18.70	0.56 [0.54 0.58]	**0** [0 0]	**0** [0 0]	**0.003**	**0.008**
	Bird 3		**0.02**±0.0003	**8.03**	**0.79** [0.78 0.79]	0.21 [0 1.04]	0.21 [0 1.04]	0.006	0.033
	Bird 5		0.02±0.007	23.39	0.11 [−2.23 0.80]	1.27 [0 10.64]	2.29 [0 10.34]	0.012	0.019
	Bird 1	DF	**0.07**±0.01	**15.28**	**0.60** [0.39 0.72]	**0.38** [0 2.5]	7.37 [0 18.18]	0.024	0.017
	Bird 2		0.09±0.02	16.05	0.37 [0.11 0.52]	0.59 [0 5.71]	**5.32** [0 17.78]	0.033	0.020
	Bird 3		0.26±0.03	24.69	−0.03 [−0.32 0.06]	1.29 [0 10]	12.44 [2.17 27.03]	0.015	0.042
	Bird 4		0.16±0.03	20.31	0.09 [−0.23 0.32]	4.72 [0 21.43]	28.56 [7.69 62.07]	**0.011**	**0.014**

Bold indicates the best metric within each bird–muscle subgroup (four birds, one muscle).

**Fig. 7.**
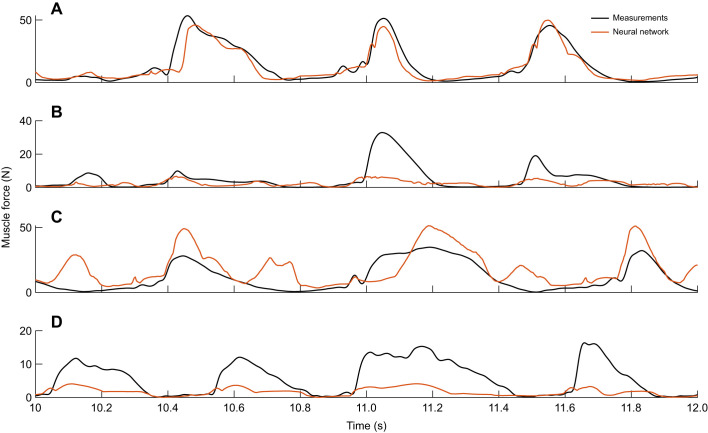
**Example muscle force predictions compared with measurements.** Predictions are shown for the best network (NN-b5) for part of a trial with speed 1.8 m s^−1^ and 7 cm elevation for the four test scenarios. (A) Same bird (bird 3), same muscle (LG). (B) Same bird (bird 3), different muscle (DF). (C) Different bird (bird 5), same muscle (LG). (D) Different bird (bird 4), different muscle (DF).

Our neural networks were able to replicate the measured force well when applied to a muscle they were trained on, while for the other muscle, they underestimated the peak force (Fig. 7 for network NN-b1; Figs S2, S3 and S4 for other networks). In the cases where the peak force was severely underestimated (Fig. 7B; Figs S2B, S3B and S4B), the median coefficient of determination became negative. Fig. 7A shows a trial that was excluded from training, for a bird and muscle that were used for training. Here, the neural network estimations matched the measurements closely. The force was underestimated slightly for most of the visible force peaks, while the small initial peaks (e.g. at 10.35 s and 10.95 s) were estimated more smoothly than in the data. For the predictions on the LG muscle of a different bird, the peak forces were overestimated (10.45 s, 11.2 s and 11.82 s) and smaller, trailing peaks were generated (10.7 s and 11.15 s), despite no such peaks being evident in the measurements (Fig. 7C). For the DF, which was not used in training, the neural network underestimated the peak force by at least 6 N for the unseen bird (bird 4) (Fig. 7D) and more than 20 N for bird 3 (Fig. 7B). Furthermore, for bird 3, the timing of the force did not match, as the force fall time was longer (Fig. 7B and Table 2). Despite the force underestimation for the DF of bird 4, the estimation of the force rise and fall times was more accurate compared with those on the DF of other birds (Fig. 7D and Table 2).

**Fig. 8.**
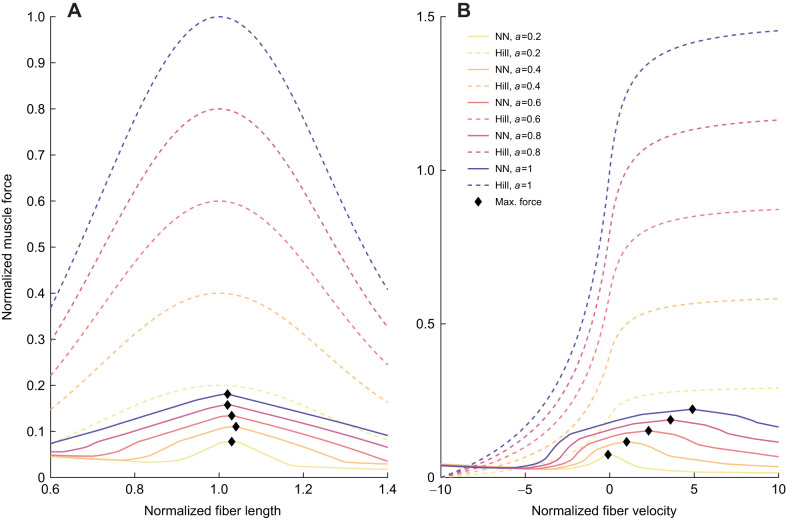
**Force–length and force–velocity relationships for NN-b5 and a standard Hill-type muscle model.** (A) Force–length relationship including parallel elastic element. (B) Force–velocity relationship. *a*, muscle activation.

The neural networks could not reproduce the force–length and force–velocity relationships well (Fig. 8 for NN-b5; Fig. S5 for other networks), which can also explain the underestimation of the DF force. The maximum normalized muscle force was about 0.18 and 0.22 for the force–length and force–velocity relationships, respectively, which indicates that the maximum muscle force was never achieved. This result indicates that the normalization to the average maximum measured activation in the processed data might not have been equivalent between the DF and LG, which explains the underestimations seen in Figs 5 and 7. The characteristic shape of the force–length relationship was maintained at all activation levels, albeit with a more moderate slope, with the maximum force consistently observed very close to optimal fiber length (1.03, 1.04, 1.03, 1.02 and 1.02, in order of increasing activation). The force–velocity relationship showed a decrease in muscle force generation with increasing shortening velocities, which plateaued around 0.03 for a shortening velocity larger than −6 fiber lengths per second. The increased force generation during lengthening was achieved at a fraction of the standard force–velocity curves. With increasing activation, the maximum normalized force shifted to higher lengthening velocities, reaching 0.22 at maximal activation (Fig. 8). For NN-b1 and NN-b3, the maximum normalized force shifted to increasingly longer fiber lengths with increasing activation, while for NN-b2 the maximum was again maintained around optimal fiber length. However, the standard bell-shaped curve was not observed for any of the neural networks. For NN-b1 and NN-b3, increasing activation led to the maximum normalized force at higher shortening velocities in the force–velocity relationship (Fig. S5).

## DISCUSSION

We trained neural networks to estimate muscle force from activation, CE length and CE velocity and showed that they generally outperform optimized Hill-type muscle models and that they estimate force more accurately on the same muscle of another individual than on another muscle of the same individual. The mRMSE was lower for the neural networks than for the Hill-type muscle models for all test scenarios, while the median coefficients of determination were higher and mRMSE for missed or extra predicted peaks was lower, but Hill-type models predicted the rise time more accurately (Table 1). There was no evidence of overfitting to the optimization data in the Hill-type models that could explain this poorer performance. Some results pointed to overfitting to training data for the smaller dataset, because the difference between training and validation loss was bigger for the smaller dataset than for the larger one. Furthermore, using the smaller dataset, the mean coefficient of determination was highest for the bird and muscle used for training, which was not necessarily the case when using a larger dataset. We found that the neural networks generally estimated force more accurately (lower mRMSE, mean relative RMSE, higher median coefficient of determination and lower mRMSE on force peak detection, and force rise and fall times) when applied to the same muscle as used for training but on a different individual, compared with estimations on a different muscle of the same individual as used for training (Table 2, Figs 5 and 7; Figs S2, S3 and S4). Furthermore, we found that our networks generally underestimated the maximum force amplification in the force–length and force–velocity relationships, but that they represented the force–length relationship well at low activation levels, while most neural networks represented the force–velocity relationship well primarily for shortening. These results indicate that the networks did not perform well outside the training data distribution (Fig. 8; Fig. S5).

Our results show the potential of using neural networks to estimate muscle forces from muscle activation, muscle length and muscle velocity. We showed that our neural networks outperform Hill-type muscle models even on unseen muscles and individuals (Table 1). However, we did not test our models across species, which is a crucial next step in order to create muscle models from neural networks using *in vivo* data. The advantage of a neural network is that it does not depend on pre-defined assumptions, which is the case for current muscle models such as the recent VEXAT model, developed by Millard et al. (2024). Another practical advantage of neural networks is their speed. Even though neural network training may be lengthy, their inference is fast and thus computationally inexpensive (Rane et al., 2019). Additionally, by training neural networks on datasets that were recorded in dynamic conditions, it is ensured that they better capture dynamic muscle behavior compared with Hill-type muscle models, which are based on limited, static conditions. Capturing such slight nuances, regardless of species, offers valuable insight into *in vivo* muscle behavior. These improved estimations can be observed when neural networks estimate the smaller peaks that occur just before large peaks (Fig. 7C at 10.3 and 10.95 s, Fig. S2C at 10.3 and 11.6 s, and Fig. S4C at 8.95 and 10.2 s), which are generally not captured by Hill-type muscle models. These muscular intricacies will be especially important in faster movements, such as running or change-of-direction movements, where the dynamic behavior of muscles is more pronounced (De Groote et al., 2016).

The reduced accuracy of the DF muscle predictions, as well as the underestimation of the maximum force amplification in the force–length–velocity relationships could be explained by inaccuracies in the maximum isometric force, in the activation scaling, or in the activation–deactivation time constants. Our dataset did not include tasks with maximal activation for normalization. Instead, we used the average of peak measured activation over the highest-speed (submaximal) trials, which might have led to a different normalization in the different muscles. This different normalization in turn might have led to force underestimations and worse median coefficients of determination, when the network was applied to another muscle (Fig. 5, Fig. 7; Figs S2, S3 and S4). However, the negative median coefficients of determination, indicating that the average force measurement would be more accurate than the predictions, appear to be largely individual and muscle specific (i.e. the DF muscle of birds 3 and 4). The difference in normalization could also have led to the reduced maximum force amplification in the force–length–velocity relationships. For both of these relationships, the amplification factor was less than 0.25, instead of 1 for the force–length and above 1 for the force–velocity relationship (Fig. 8). These low amplification factors could indicate that the activation scaling used was not equivalent between the LG and the DF, or that the maximum isometric force was not accurately estimated.

Our neural networks did not match the shape of the static force–length and force–velocity relationships either (Fig. 8; Fig. S5). For two network types, increasing activation did not shift the normalized maximum force away from the optimal fiber length (Fig. 8; Fig. S5B), but flatter curves were observed. For the remaining two network types, the optimal fiber length increased with increasing activation, while previous experiments have shown that it decreases with increasing activation (e.g. Roszek and Huijing, 1997; Rack and Westbury, 1969; Schwaner et al., 2024). Nevertheless, a common observation between our most efficient network and those of Schwaner et al. (2024) is that at the highest activation, the force–length curve tends to flatten out. Furthermore, we found that the maximum shortening velocity decreased with decreasing activation, which was previously shown (Chow and Darling, 1999). However, the networks' force–length–velocity relationships did not display the correct shape for the entire tested range. The optimal fiber length was consistently around 1 when activation was low. For two network types, when activation increased, the optimal fiber length shifted to longer lengths. Similarly, for the majority of network types, the force–velocity relationship did not display a force increase at lengthening velocities, though the shape for shortening velocities was relatively accurate. These results indicate that the networks' output was inaccurate both at the extremes of and outside the training data distribution, e.g. at large activation and large lengthening velocities (Fig. 8; Fig. S5). This inaccuracy could be explained by a lack of data in these regions as most data were recorded at low and medium activation, and fiber lengths and velocities were closer to optimal and isometric conditions. Therefore at this stage, using a neural network to model muscle performance for movements whose eccentric phase typically requires maximal activation and large lengthening velocities, such as sprinting, change of direction, jump landing or descending stairs, would at the very least be inefficient. Another explanation for the inaccuracies in the force–length–velocity relationships could be the *in vivo* nature of our data, because, as Schwaner et al. (2024) observed, the *in vivo* muscle operating range does not align with the force–length plateau measured *in situ*. Still, understanding the force–length–velocity relationships uncovered by the neural networks could potentially lead to valuable insights about muscle mechanics and performance outside the training distribution. Thus, our findings reinforce the need for more information on the force–length–velocity relationships under sub-maximal or transient conditions.

To achieve better neural network performance, we recommend creating a more suitable training dataset. An ideal dataset would incorporate a broad range of activities and species. It would include training data of at least three different muscles of different fiber type (i.e. predominantly slow or fast twitch), at least three different animal species, to capture a broader – though still not exhaustive – range of muscle diversity, and different tasks that represent the full functional range of the animals as much as possible. By including more muscles in the training, we might avoid a network bias towards the single muscle used in training. Such a bias is likely to be present in our networks, which performed better on the muscle that they were trained on (LG) than on the unseen muscle (DF). Furthermore, to apply such a network to a human musculoskeletal model, we would ideally validate it on human data. Such a validation is difficult as it typically requires invasive sensors. However, shear-wave elastography (for muscle–tendon force data) along with ultrasound data (for fascicle lengths) and surface electromyography (for activation) could be used to generate a human dataset (Martin et al., 2018). Still, validating the model on different species before applying it to human data would verify the lack of any species-specific network bias. To include the full functional range, the ideal dataset should include both submaximal and near-maximal tasks. These near-maximal tasks should ensure that the normalization is comparable between muscles, while the submaximal tasks cover normal muscle activation during movement.

It is also important to realize that a neural network is only as good as the data used for its training. In practice, training datasets will always have limitations and ethical constraints. One practical implication is the development of muscle fatigue, which distorts the relationship between activation and force over time, thus making the collection of high-quality, standardized, *in vivo* muscle data challenging. Therefore, it might not be possible to include all activities that are required to comprehensively cover all movements. Furthermore, there might be too much variability in data recordings between species and muscles to train a neural network that is applicable on all species and muscles from one large dataset. Therefore, a neural network might not be as flexible as a Hill-type muscle model, though Hill-type muscle models also often approximate parameters that have not been directly measured for all muscles, and are used despite known inaccuracies (e.g. Abbott and Aubert, 1952). However, the internal relationships of a Hill-type muscle model are understood, and it is therefore known how they approximate muscle mechanics. In contrast, we generally do not understand the internal behavior of a neural network, which acts like a black box, and therefore a neural network cannot be deployed without proper validation. Nonetheless, DeepLabCut, an effective machine learning model for pose estimation, has a similar black box behavior. This model can be used for different movements as well as different species through transfer learning with little additional training data (Nath et al., 2019). Such an approach could potentially also be used to transfer muscle models between species. However, transfer learning towards humans would again be challenging as direct measurements are difficult. Alternatively, optical motion capture, possibly combined with surface electromyography, could create sufficient training data. However, this approach normally estimates muscle forces using inverse kinematics, dynamics and a Hill-type muscle model. So in this case, it is possible that the network will learn to replicate a Hill-type muscle model instead of the actual muscle mechanics. Furthermore, the black box nature could be reduced through physics-informed training (e.g. Raissi et al., 2019; Zhang et al., 2022). In physics-informed training, the loss is calculated not only from training data but also based on some physics relationships that the network should follow. For example, in the case of a muscle model, a loss could be included based on how well the network follows the force–length and force–velocity relationships. Then, the neural network is not forced to follow those relationships exactly, but uses them to steer the training, while still also matching the training data. To achieve that, caution should be exercised such that a delicate balance is found between physics-based and data-based learning, given our findings which support that neural networks do not inherently detect these assumed relationships from *in vivo* dynamic measurements.

We also compared our results with other relevant muscle models (Whitney et al., 2022; Liu et al., 1999). Liu et al. (1999) successfully trained an artificial neural network to estimate muscle force in a cat soleus solely from electromyography signals (Liu et al., 1999). We compared our mean relative RMSE values with theirs in the most similar shared scenario: training on EMG and force data of the same muscle of several animals (cats or guinea fowl) and testing on another (cat or guinea fowl). Comparing the mean relative RMSE on the LG muscle of unseen birds for all four of our networks with their relative RMSE at a speed of 1.2 m s^−1^ (the highest speed of their dataset that is closest to the lowest speed of our dataset), we observed comparable and slightly higher relative RMSE values (17.9%, 14.97% and 14.37% compared with 12% in table 3 of Liu et al., 1999), which could be explained by the greater complexity and variability of the input space of our model (i.e. more animals and more input variables were used for training). Predictions on the LG of bird 5 using NN-b5 resulted in worse relative RMSE (23.39% compared with 12% in table 3 of Liu et al., 1999). However, predictions on bird 5 by NN-b5 were considerably worse than for other individuals, despite this network type being the most accurate when considering all metrics. When we used our data to train a model to estimate force from electromyography data only, the network performance was worse. This performance drop could be caused by the fact that our dataset, which included perturbations, was specifically designed to include cases where a different force was created despite having the same muscle activation, while Liu et al. (1999) used steady-state walking data, where the relationship between muscle activation and force is more direct. Because of this training approach, their model will likely not generalize to non-steady conditions, as these are not included in their training data. We also compared our median coefficient of determination values with a recently developed winding filament model (Whitney et al., 2022), which was optimized on the same data. Compared with this model, our median coefficients of determination were typically lower [the range for the LG muscle was between 0.11 and 0.79 compared with 0.71 and 0.81 in Whitney et al. (2022), while for the DF muscle it was between −0.03 and 0.60 compared with 0.48 and 0.82]. However, Whitney et al. (2022) performed a model parameter optimization for each muscle and each trial individually, while we tested on unseen birds or muscles.

As the dataset used here was not recorded specifically for neural network training, we could not test additional approaches. For example, we suspect that the underestimation and overestimation of peak forces in the DF is related to a difference in activation scaling between the two muscles or inaccurate estimates of the maximum isometric force. We scaled the activation to the average peak activation recorded in the respective muscle over the highest-speed trials of the same bird, but it is very possible that this is not equivalent between the muscles if less activation is required in one muscle than in the other for the recorded activities. Furthermore, the maximum isometric force was determined from the physiological cross-sectional area through the maximum stress, but the maximum stress might be different between muscles (Maganaris et al., 2001). As the network is only trained on one muscle, it has likely developed a bias towards this muscle's error in the activation and maximum isometric force, and therefore the mRMSE and the mean relative RMSE are much lower for the LG than for the DF. Furthermore, this bias seems persistent among the same muscle of different individuals, but not between different muscles of the same individual. Using training data from a more varied selection of muscles should improve neural network performance.

Many different avenues can be explored with a dataset of dynamic muscle behavior, such as further development of Hill-type muscle models or exploring different model architectures. Currently, Hill-type muscle model parameters are often based on experiments with controlled conditions in more static scenarios, but they can also be fitted to dynamic experiments, as shown in this paper. A Hill-type muscle model with parameters estimated from dynamic data might better represent muscle mechanics in dynamic situations than one with parameters from a controlled-condition experiment. Furthermore, dynamic datasets could also help clarify the underlying mechanisms of the force–length and force–velocity relationships during sub-maximal and transient conditions. Different model equations could be explored using a dynamic dataset, or the relationship can be extracted from the data, as we have done with our machine learning approach. Another avenue is to explore advanced neural networks. Recurrent neural networks could capture the history dependence in muscle force generation (Herzog, 2004), thereby improving network performance along with the representation of muscle mechanics. By comparing the quality of different architectures or input combinations, the relevance of different muscle states or parameters to muscle force generation could be explored to formulate hypotheses about muscle mechanics. These hypotheses could then be further tested in specific experiments.

In conclusion, we found that a neural network can estimate muscle force more accurately than a Hill-type muscle model, on both different muscles and different individuals. However, if estimation of the force rise and fall times is the primary goal, Hill-type models can yield better or equally accurate results, respectively. Training neural networks on a larger dataset seemed to reduce overfitting towards the training data. We also found that when training on data of one muscle, neural networks make better estimations on the same muscle of another individual, than on another muscle of the same or a different individual. Future studies should further investigate whether this effect can be mitigated by training on multiple muscles, which reduces the bias towards the muscle used for training, while we have also made recommendations on an ideal dataset including maximum activation tasks which could also mitigate issues of activation scaling. In conclusion, our findings support that neural networks can be a promising approach to new muscle model implementations, in place of other models currently in use (i.e. Hill-type muscle models), and they could also be a useful tool for generating new hypotheses about muscle mechanics.

## Supplementary Material

10.1242/jexbio.250268_sup1 Supplementary information
