# Supplementary material for: Neural networks estimate muscle force in dynamic conditions better than Hill-type muscle models
Source: J Exp Biol. 2025 Nov 18;228(22):jeb250268. doi: 10.1242/jeb.250268 (PMC12669831; doi:10.1242/jeb.250268)
Supplement: Supplementary information [file jexbio-228-250268-s1.pdf]

This supplemental document shows the loss curves of the best neural network type trained on a large dataset (NN-b5), and Fig. 7 and 8 for the networks NN-b1, NN-b2 and NN-b3.

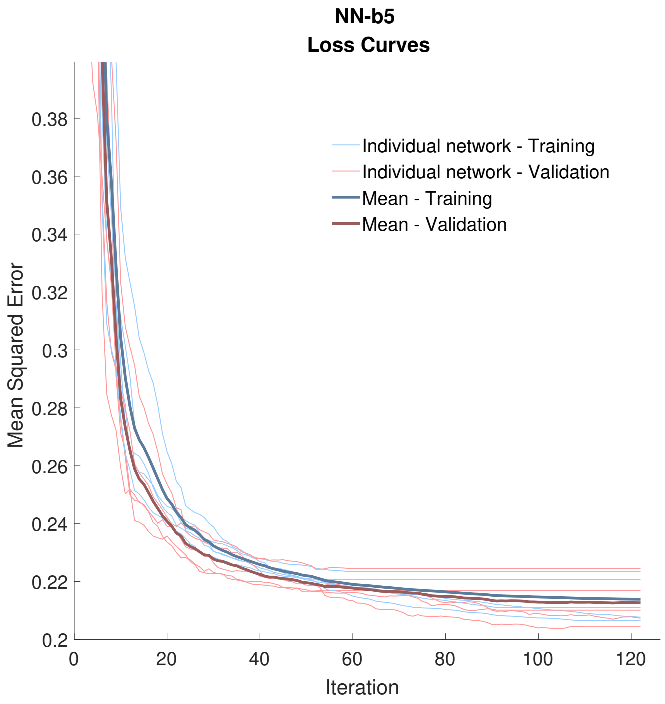

Fig. S1. Training and validation losses during training of neural network type NN-b5. The thin blue and red lines show the training and validation loss trajectories of individual NN-b5-type neural networks. The darker single blue and red lines show the average trend for each respective loss.

Journal of Experimental Biology • Supplementary information

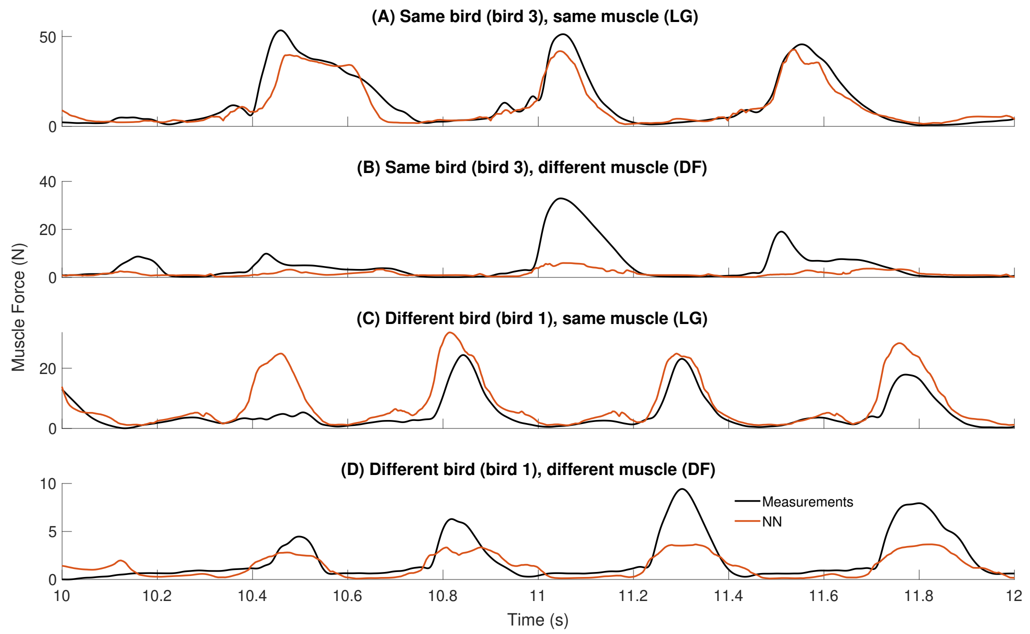

Fig. S2. Muscle force predictions by NN-b1 and measurements over time, for part of a trial with speed 1.8 m s^{-1} and 7 cm elevation for (A) the same bird and muscle as used for network training,(B) the same bird but a different muscle, (C) the same muscle of a different bird, and (D) a different muscle of a different bird.

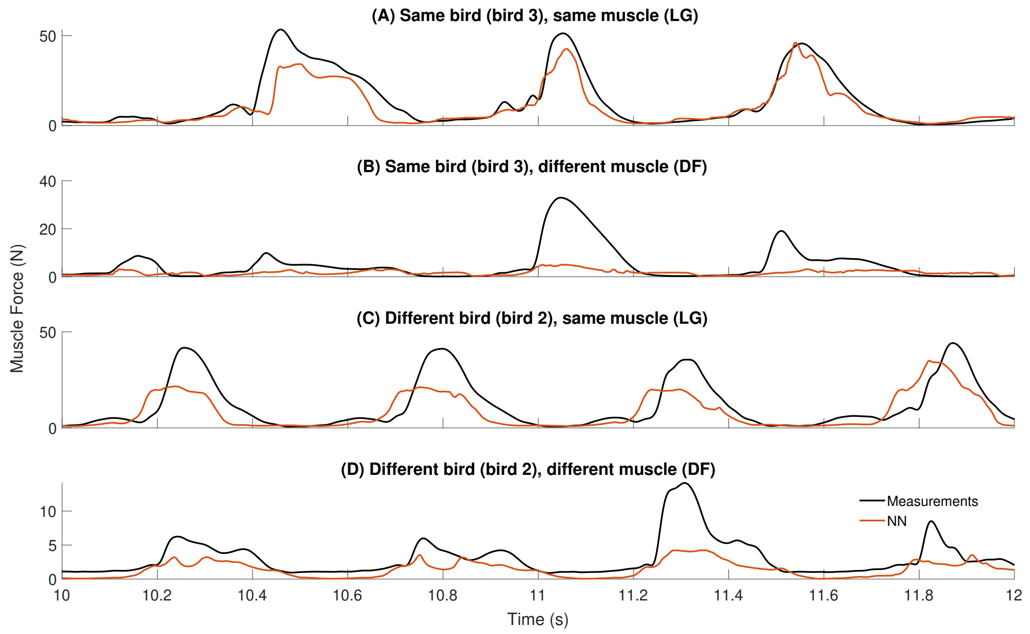

Fig. S3. Muscle force predictions by NN-b2 and measurements over time, for part of a trial with speed 1.8 m s^{-1} and 7 cm elevation for (A) the same bird and muscle as used for network training,(B) the same bird but a different muscle, (C) the same muscle of a different bird, and (D) a different muscle of a different bird.

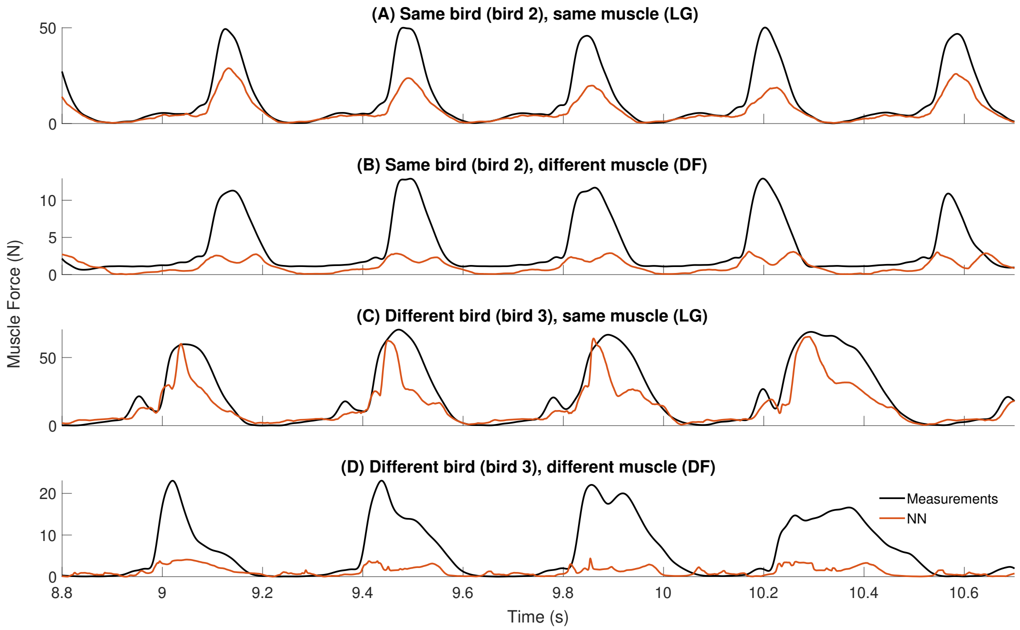

Fig. S4. Muscle force predictions by NN-b3 and measurements over time, for part of a trial for (A) the same bird and muscle as used for network training, (B) the same bird but a different muscle, (C) the same muscle of a different bird, and (D) a different muscle of a different bird. The presented trials do not include an obstacle and their speeds are 3.8 and 3.5 m s^{-1}, for bird 2 and 3, respectively. Despite their speed difference, these trials allowed for the closest comparison of test scenarios, among the reserved test trials.

Journal of Experimental Biology • Supplementary information

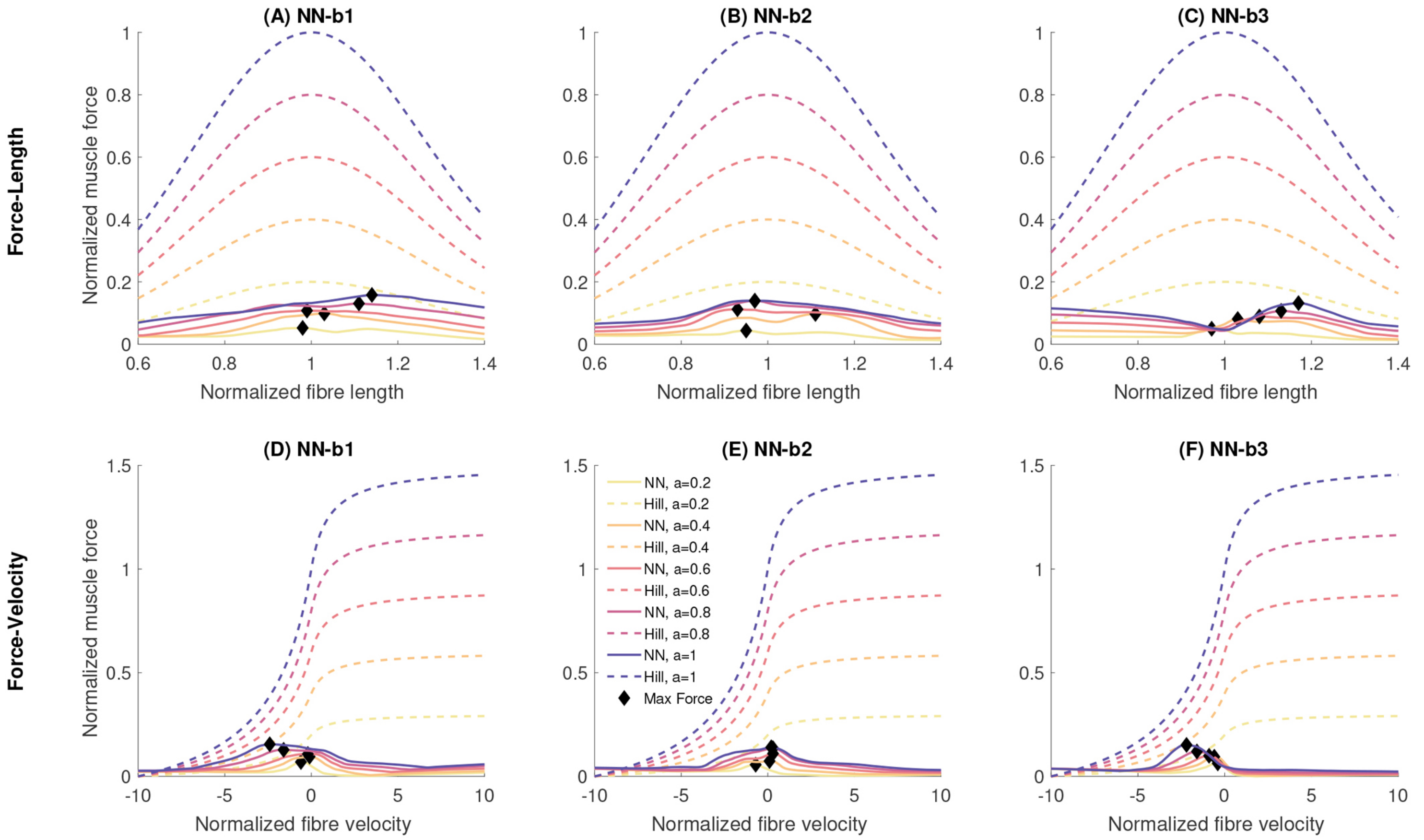

Fig. S5. Force-length relationships, including parallel elastic element, and force-velocity relationships for NN-b1 ((A) & (D)), NN-b2 ((B) & (E)), and NN-b3 ((C) & (F)).

Journal of Experimental Biology • Supplementary information
